# Associations between temperament dimensions and dental anxiety in parents of the FinnBrain Birth Cohort Study

**DOI:** 10.1111/eos.12897

**Published:** 2022-10-27

**Authors:** Juuso Arkkila, Auli Suominen, Saara Nolvi, Kari Rantavuori, Hasse Karlsson, Linnea Karlsson, Satu Lahti

**Affiliations:** ^1^ Department of Community Dentistry University of Turku Turku Finland; ^2^ Turku Institute for Advanced Studies Department of Psychology and Speech‐Language Pathology University of Turku and Turku University Hospital Turku Finland; ^3^ FinnBrain Birth Cohort Study, Department of Clinical Medicine, Turku Brain and Mind Center University of Turku Turku Finland; ^4^ Department of Pediatric Dentistry and Orthodontics University of Turku Turku Finland; ^5^ Cleft Palate and Craniofacial Center, Department of Plastic Surgery Helsinki University Hospital and Helsinki University Finland Helsinki Finland; ^6^ Centre for Population Health Research University of Turku Turku Finland; ^7^ Department of Psychiatry University of Turku and Turku University Hospital Turku Finland

**Keywords:** anxiety, dental fear, emotions, personality, temperament

## Abstract

We evaluated associations between dental anxiety and four temperament dimensions: effortful control, extraversion/surgency, negative affect and orienting sensitivity among 2558 parents in the FinnBrain Birth Cohort Study. Dental anxiety was measured with the Modified Dental Anxiety Scale, and temperament with the Adult Temperament Questionnaire. Associations between dental anxiety and temperament dimensions were modelled using linear and logistic (cut‐off ≥ 19 for high dental anxiety) regression analyses adjusting for general anxiety and depressive symptoms, age and education. In women and men, dental anxiety was positively associated with negative affect (women β = 1.10; 95%CI 1.06–1.15; men β = 1.11; 95%CI 1.05–1.18) and negatively associated with effortful control (women β = 0.95; 95% CI0.92–0.99, men β = 0.90; 95% CI 0.85–0.95). In women, extraversion/surgency was also positively associated with dental anxiety (β = 1.04; 95%CI 1.00–1.08). For high dental anxiety, negative affect in women (OR = 2.00; 95%CI 1.31–3.06) and men (OR = 5.21; 95%CI 1.72–15.83) and for extraversion/surgency in women (OR = 1.50; 95%CI 1.01–1.47) associated positively with dental anxiety, but for effortful control, the association was not statistically significant. Dentists should understand that temperament dimensions affect the risk for dental anxiety more strongly than general anxiety or depressive symptoms. Dimensions negative affect and extraversion/surgency may increase and effortful control decrease the risk.

## INTRODUCTION

One third of Finns are dentally anxious; one in ten are highly dentally anxious, and women more often so than men [1]. High dental anxiety is associated with irregular dental attendance, which may in turn lead to poor oral health and poor oral health‐related quality of life [2, 3, 4, 5, 6]. Dental anxiety has been shown to be associated with psychological symptoms, especially with general anxiety [7, 8, 9, 10, 11, 12, 13, 14, 15, 16, 17, 18, 19, 20] and depression [7, 8, 15, 16, 19, 20]. Together with personality and other traits, such as alexithymia [21, 22, 23], these have been referred to as conferring ‘constitutional vulnerability to dental anxiety disorders’ [24, 25, 26] and might reflect endogenous origin of dental anxiety [18].

Temperament is a core aspect of personality that emerges early in life [27]. It is rooted in biological systems underlying the individual's emotional reactivity and attentional capacities [28]. In adults, the following core temperament dimensions have been described: negative affect referring to proneness for negative emotions such as fear, sadness and frustration; effortful control, which refers to effortful self‐regulation and control of behaviors (e.g., inhibitory control, effortful attention); extraversion/surgency referring to a tendency for positive affect and sociability; and orienting sensitivity, referring to affective and general perceptual sensitivity and associative sensitivity [28]. These dimensions overlap considerably with personality traits, such as extraversion, agreeableness, intellect/openness, conscientiousness and neuroticism [28]. Psychological symptoms and disorders, such as general anxiety and depression, are reportedly associated with certain temperament and personality traits, especially harm avoidance, negative affect/neuroticism and poorer self‐regulation and self‐directedness [29, 30].

In children, multiple dimensions of temperament, such as negative emotionality, shyness and impulsivity, have been positively correlated with dental anxiety whereas others such as sociability, a trait typically linked with extraversion, had negative correlation with dental anxiety [31, 32]. However, surprisingly, in adults, there is little research on the association between dental anxiety and temperament or personality. Traits such as novelty seeking, a trait corresponding to the temperament trait of surgency/extraversion, and traits of openness, shyness, impulsivity and emotionality have shown to correlate positively with dental anxiety [33, 34]. In turn, higher neuroticism, a personality trait similar to negative affect, correlated positively with dental anxiety, whereas extraversion was negatively linked with dental anxiety among university students; the associations also differed by gender [35]. The existing studies on temperament have been conducted in dental phobics [34] or very small [33] unrepresentative samples, and findings from large general population samples are lacking. Thus, the aim of the study is to study whether and how adult temperament traits are associated with dental anxiety in a general population of Finnish parents of young children.

## MATERIAL AND METHODS

This was a cross‐sectional study using data from the FinnBrain Birth Cohort Study (finnbrain.fi), a multidisciplinary study on the effects of environmental and genetic factors on child brain development and health [36]. Participants were parents who were recruited after ultrasonography appointments that are offered free of charge around gestational week 12 for every pregnant mother in Finland. Recruitment took place in municipal maternity clinics in the Hospital District of Southwest Finland and the Åland Islands in Finland in 2011−2015. The Ethics Committee of the Hospital District of Southwest Finland approved the study protocol (14.6.2011 ETMK:57/180/2011 § 168). Of those informed about the study (*N* = 5790), a total of 3808 (66%) mothers and 2623 fathers or other partners of the mother, expecting 3837 children (twins included) agreed to participate, and of those, 3095 (81%) mothers and 2011 (77%) fathers returned the baseline questionnaire and started the study [36]. Both parents provided written informed consent.

Data on dental anxiety were collected 24 months after delivery, and data on temperament 12 months after delivery. For this study, parents from whom data on both dental anxiety and temperament were available were included in the analyses, resulting in a total of 1106 (19%) mothers and 544 fathers. Information on the covariates education and father's age were collected at gestational week 14, while mother's age was obtained from the Finnish Medical Birth Registry. The data on general anxiety and depressive symptoms were collected 24 months after delivery; that is, concurrently with the information on dental anxiety.

Dental anxiety was measured with the Finnish translation of the Modified Dental Anxiety Scale (MDAS). The MDAS has shown acceptable validity (concurrent and discriminant) and high internal consistency [37, 38, 39]. The questions in the MDAS were: (1) if you went to your dentist for treatment tomorrow, how would you feel; (2) if you were sitting in the waiting room (waiting for treatment), how would you feel; (3) if you were about to have a tooth drilled, how would you feel; (4) if you were about to have your teeth scaled and polished, how would you feel; and (5) if you were about to have a local anesthetic injection in your gum, above an upper back tooth, how would you feel? Each question had five response options, ranging from 1 (not anxious) to 5 (extremely anxious). For the MDAS, a sum score with a possible range 5–25 was calculated. The MDAS was also dichotomized to ‘no to moderate dental anxiety’ (5–18) and to ‘high dental anxiety’ (19–25) as suggested in a previous study [37].

Temperament was measured using the Finnish version of the Adult Temperament Questionnaire (ATQ), a validated 77‐item instrument for assessing adult temperament [28]. Participants were asked to assess how well each item described them on a scale (from 1 to 7), with a higher score reflecting a higher level of the characteristic in question. The four standard main dimensions of ATQ, effortful control, extraversion/surgency, negative affect and orienting sensitivity, were used in the present study. In women, the Cronbach's alphas for the dimensions were 0.83, 0.79, 0.72, 0.77, for effortful control, extraversion/surgency, negative affect and orienting sensitivity, respectively. The corresponding alphas among men were 0.85, 0.79, 0.76 and 0.77, respectively.

General anxiety was measured with the Finnish version of the Symptom Checklist‐90 (SCL‐90, anxiety subscale with Cronbach's alpha = 0.89) [40, 41, 42]. The 10 SCL‐90 items were scored on a 5‐point Likert scale (from 0 to 4) and summed up to total score ranging 0–40. Depressive symptoms were measured with the Finnish version of the Edinburgh Postnatal Depression Scale (EPDS), having mostly high sensitivity and specificity, and being valid for screening both pre‐ and postnatal depressive symptoms [43, 44], also among fathers [45, 46]. The 10 questions EPDS were scored on a 4‐point Likert scale (from 0 to 3) and summed up to total score ranging 0–30.

If there were ≤30% missing items for the ATQ, EPDS, MDAS or SCL‐90 (anxiety subscale) they were imputed with the mean value of the answered items. Education was categorized to three levels, low (high school/vocational ≤ 12 years), medium (polytechnics/applied university), and high (university degree). Sex differences in the MDAS scores, temperament dimensions, SCL‐90 scores, EPDS scores and age were tested with the Mann‐Whitney U tests and for education sex differences were tested with Pearson Chi‐square tests. The bivariate associations between MDAS sum, ATQ dimensions, age, SCL‐90 and EDPS were assessed with Spearman's correlation coefficients separately for women and men. The bivariate associations between dichotomized MDAS, temperament dimensions and education were assessed with Mann‐Whitney U‐test separately for women and men. The covariates were selected based on previous results [1‐20, 29–30]. Finally, the associations between all temperament dimensions and dental anxiety at the same model (with cut‐off for high dental anxiety (MDAS ≥ 19) in logistic regression) were modelled using linear and logistic regression analyses adjusted for general anxiety and depressive symptoms, education (as dummy variable in linear regression) and age, separately for women and men. All analyses were conducted using SPSS (IBM). Alpha was set at 0.05 (2‐sided).

## RESULTS

Table 1 summarizes the sample characteristics. The prevalence of high dental anxiety was 7.4% (*n* = 83) in women and 3.1% (*n* = 15) in men. Women scored higher in dental anxiety as well in temperament dimensions, with the exception of extraversion/surgency.

**TABLE 1:** Age, education, general anxiety, depressive symptoms, dental anxiety and four temperament dimensions by sex

	Women			Men			
	*n*	Mean	SD	*n*	Mean	SD	*P*
MDAS	1113	10.6	4.6	547	8.8	3.7	<0.001^a^
ATQ NA	1113	4.0	0.7	547	3.2	0.7	<0.001^b^
ATQ EC	1112	4.7	0.7	547	4.8	0.7	<0.001^b^
ATQ ES	1111	4.7	0.7	547	4.6	0.7	0.084^b^
ATQ OS	1108	4.6	0.8	547	4.2	0.7	<0.001^b^
SCL‐90	1113	2.8	3.9	547	2.5	3.8	0.060^a^
EPDS	1112	4.5	4.2	547	3.8	3.8	0.002^a^
Age	1113	31.1	4.3	547	33.3	5.3	<0.001^b^
Education	*n* (%)			*n* (%)			
Low	313 (28.1)			203 (37.1)			
Medium	318 (28.6)			158 (28.9)			<0.001^c^
High	482 (43.3)			186 (34)			

MDAS = Modified Dental Anxiety Scale.

ATQ NA = Negative affect, ATQ EC = Effortful control, ATQ ES = Extraversion/Surgency, ATQ OS = Orienting sensitivity.

SCL‐90 = Symptom Checklist −90 (anxiety subscale), EPDS = Edinburgh Postnatal Depression Scale.

Categorization for Education: Low = high school/vocational ≤ 12 years, Medium = polytechnics/applied university, High = university degree. *P*‐value for ^a^ Mann‐Whitney U‐test, ^b^ t‐test, ^c^ Chi square test.

In bivariate analyses, modest to moderate correlations between temperament dimensions, dental anxiety, general anxiety and depressive symptoms were detected. Negative affect correlated positively (r = 0.207; r = 0.264) and effortful control negatively (r = −0.191; r = −0.305) with dental anxiety among both women and men, respectively. Depressive symptoms correlated positively with dental anxiety (r = 0.110; r = 0.197) and individual's level of negative affect (r = 0.295; r = 0.264), and negatively with level of effortful control (r = −0.318; r = −0.374) and extraversion/surgency (r = −0.156; r = −0.198), among women and men, respectively.

When comparing those with high dental anxiety to those with no or low dental anxiety, associations with temperament dimensions were similar for negative affect and effortful control. Those who reported high dental anxiety also reported higher negative affect (mean = 4.3, *P* < 0.001 for women and mean = 3.8, *P* = 0.011 for men) and lower effortful control (mean = 4.5, *P* = 0.019 for women and mean = 4.4, *P* = 0.006 for men) than those who reported no or moderate dental anxiety (means for negative affect = 3.9 and 3.2 and means for effortful control = 4.7 and 4.8, for women and men, respectively). However, in women, those with high dental anxiety reported also higher orienting sensitivity (mean = 4.8, *P* = 0.043) than those with no or low dental anxiety (mean = 4.7).

When adjusting for general anxiety and depressive symptoms, age, and education, higher dental anxiety was associated with higher levels of negative affect and lower levels effortful control both among men and women (Table 2). In women, there was also a positive, but weak association between higher extraversion/surgency and higher dental anxiety. Notably, the association between dental anxiety and general anxiety and depressive symptoms was no longer significant with temperament in the model.

**TABLE 2:** Association between dental anxiety and four temperament dimensions (multiple linear regression) adjusted for general anxiety (SCL‐90 anxiety subscale) and depressive (EPDS) symptoms, age and education

	MDAS total					
	Women			Men		
	*β*	95% CI	*P*	*β*	95% CI	*P*
ATQ NA	1.104	(1.058, 1.152)	<0.001	1.113	(1.049, 1.181)	<0.001
ATQ EC	0.954	(0.917, 0.993)	0.020	0.902	(0.852, 0.954)	<0.001
ATQ ES	1.039	(0.999, 1.08)	0.056	1.042	(0.993, 1.093)	0.091
ATQ OS	0.995	(0.963, 1.028)	0.770	0.998	(0.956, 1.043)	0.942
SCL‐90	1.000	(0.998, 1.012)	0.996	1.010	(0.944, 1.014)	0.086
EPDS	1.005	(0.992, 1.008)	0.191	0.996	(0.987, 0.999)	0.449
Age	0.985	(0.980, 0.991)	<0.001	0.993	(0.999, 1.022)	0.019
Education	0.966	(0.938, 0.994)	0.020	0.979	(0.984, 1.007)	0.240

MDAS = Modified Dental Anxiety Scale.

ATQ NA = Negative affect, ATQ EC = Effortful control, ATQ ES = Extraversion/Surgency, ATQ OS = Orienting sensitivity.

SCL‐90 = Symptom Checklist −90 (anxiety subscale), EPDS = Edinburgh Postnatal Depression Scale, Education: Low = high school/vocational ≤ 12 years, Medium = polytechnics/applied university, High = university degree.

When looking at temperament dimensions associated with the cut‐off of high dental anxiety, the findings were slightly different. Among both men and women, those with high dental anxiety had higher levels of negative affect and, among men, the association was more than twice as strong as among women. However, in women, those with high dental anxiety also had higher extraversion/surgency. Effortful control was not associated with high dental anxiety in either men or women (Table 3).

**TABLE 3:** Multiple logistic regression model on the association of four temperament dimensions with high dental anxiety, adjusted for anxiety (SCL‐90) and depressive (EPDS) symptoms, age and education, presenting odds ratios (OR) with 95% confidence intervals (95% CI) and *P*‐values

	Women			Men		
	OR	95% CI	*P*	OR	95% CI	*P*
ATQ NA	2.00	1.31−3.06	0.001	5.21	1.72−15.83	0.004
ATQ EC	1.00	0.68−1.47	0.999	0.88	0.34−2.27	0.794
ATQ ES	1.50	1.01−2.23	0.043	1.76	0.71−4.33	0.220
ATQ OS	1.08	0.78−1.50	0.637	1.32	0.62−2.80	0.476
SCL‐90	0.98	0.91−1.06	0.586	1.00	0.86−1.18	1.000
EDPS	1.04	0.97−1.11	0.311	0.95	0.77−1.17	0.640
Age	0.94	0.88−0.99	0.020	0.87	0.77−0.98	0.022
Education (ref. high)						
low	2.04	1.12−3.71	0.020	3.21	0.87−13.21	0.107
medium	1.48	0.81−2.74	0.201	0.78	0.12−5.23	0.799

High dental anxiety (1): Modified Dental Anxiety Scale MDAS = 19+ (ref. no to moderate dental anxiety: MDAS = 5−18).

ATQ NA = Negative affect, ATQ EC = Effortful control, ATQ ES = Extraversion/Surgency, ATQ OS = Orienting sensitivity.

SCL‐90 = Symptom Checklist −90 (anxiety subscale), EPDS = Edinburgh Postnatal Depression Scale, Education: Low = high school/vocational ≤ 12 years, Medium = polytechnics/applied university, High = university degree.

## DISCUSSION

In the parents of young children, those with higher negative affect reported higher dental anxiety, and those with higher effortful control reported lower dental anxiety, both in men and women. Associations were similar for those with high dental anxiety. Interestingly, among those reporting high dental anxiety, effortful control was no longer statistically significantly associated with dental anxiety, and women with high dental anxiety reported higher extraversion/surgency.

The present study has some strengths and limitations. Even though the population sample was quite large and included different socioeconomic status groups, the age and the life situation of the population sample are specific. Thus, the findings cannot be generalized to other populations. The sample included both genders, but the percentage of men was significantly lower than that for women, and the number of participants, especially males, with high dental anxiety was modest. The drop‐outs were younger, with lower education, and were more depressed and anxious than those who stayed in the study [19, 36]. These issues should be borne in mind when interpreting the findings. The strengths include reliable and validated measures, and that dental anxiety was not measured in the context of a dental visit. The 12‐month interval between the measurements and the busy life period with a young child makes the measurements vulnerable to life change. However, temperament is considered a stable feature, and dental anxiety has also been rather stable in this population over 2 years [19]. In addition, different measurement points may have reduced the chance of common method variance, which is the systematic variance shared among the variables, usually introduced by the method of measurement rather than the measures’ theoretical constructs [47].

Our findings on the association between dental anxiety and temperament dimensions of negative affect and effortful control are similar to those of previous investigations of temperament [31, 32, 33, 34] and personality traits [35] in adults. High negative affect raises the individual's tendency to react on new or unusual situations negatively or to experience more worries in general. Thus, it is likely that persons with this temperament characteristic have a higher risk of being afraid of dental situations where personal space is invaded, are potentially painful, or involve feeling a lack of control. Higher effortful control, in turn, refers to the individual's ability for better self‐regulation and self‐control. Accordingly, those with higher effortful control might better cope with situations like dental treatment, and so be less likely to develop dental anxiety in which lack of control is a key phenomenon [6].

Previous publications have frequently reported an association between negative affect and more mental health symptoms, such as higher depression and general anxiety [29, 30], which have also been associated with dental anxiety [7, 8, 9, 10, 11, 12, 13, 14, 15, 16, 17, 18, 19, 20, 21, 22, 23, 24, 25, 26, 27, 28, 29]. Interestingly, general anxiety or depressive symptoms were not associated with dental anxiety when temperament dimensions were considered simultaneously. This is a novel finding, as in previous studies not including temperament, general anxiety was consistently and depression was in most cases associated with dental anxiety [7, 8, 9, 10, 11, 12, 13, 14, 15, 16, 17, 18, 19, 20]. General anxiety and depression have also not been controlled for in previous studies of temperament and dental anxiety [33, 34, 35]. This suggests that individual traits of reacting and self‐regulation override the current mental health state. Thus, traits may be more prominent predictors of dental anxiety than mental health state, or the mental health state may be a mediator of trait‐like risk factors for dental anxiety.

Another noteworthy finding was the differences in associations between those with high dental anxiety and those with no to moderate dental anxiety when the association between effortful control and dental anxiety was no longer apparent. Though strong effortful control may in general decrease the risk of dental anxiety, this association was not observed when high‐level cut‐off of dental anxiety was in focus. Thus, very high dental anxiety may override an individual's self‐regulation capacity; a finding in line with previous research showing that very high stress or emotionally arousing stimuli may deplete self‐regulation [48]. Long‐term cortisol levels have also been associated to dental anxiety [49]. The different patterns of associations between men and women are also interesting. Women who scored higher in extraversion/surgency reported more often high dental anxiety (MDAS 19+). This is a somewhat surprising finding, since high extraversion/surgency is typically linked with a lower risk of psychological symptoms and disorders. However, the proportions of men and women with high dental anxiety were small, and so this finding should be interpreted with caution and needs to be replicated.

Our findings indicate that temperament may have a stronger role than psychological symptoms or disorders in the development of dental anxiety; the latter may have a mediating role. These findings may also help us to understand why at the same time with decreasing dental anxiety, levels the prevalence of those with high dental anxiety has remained stable [50, 51]. Understanding the role of temperament, especially negative affect and effortful control in dental anxiety has several clinical implications. Firstly, based on our findings, it may be helpful for dentists and other oral health personnel to understand that dentally anxious patient's reactions to stimuli and ability to regulate reactions may arise from temperament, a rather stable trait of the individual, instead of, for example, patient attitudes towards dental care. Secondly, after assessing patient's dental anxiety, discussing with patient on the previous reactions and emotions towards care may enhance this understanding. Thirdly, basic understanding of temperament may help dentists to utilize and further develop existing techniques to manage dental anxiety. Several of these techniques, such as building trust, providing control, using a tell‐show‐do method and relaxation strategies [52] may be especially helpful for those who have a tendency to react negatively to dental care. Adapting these techniques routinely could help to reduce dental anxiety and be particularly helpful in preventing development of dental anxiety among those with higher negative affect. Those with very high dental anxiety might need additional support, such as guidance in choosing a less stressful period in life that is best suitable for appointments to avoid excessive strain in life. For dentists, it may be helpful to understand that personality traits might override or underlie the current mental state, for example, general anxiety or depressive symptoms.

## AUTHOR CONTRIBUTIONS

**Conceptualization**: Juuso Arkkila, Satu Lahti, Saara Nolvi; **Methodology**: Juuso Arkkila, Satu Lahti, Auli Suominen; **Formal analysis**: Auli Suominen; **Investigation**: Hasse Karlsson, Linnea Karlsson; **Writing – original draft**: Juuso Arkkila, Satu Lahti, Auli Suominen; **Writing – review and editing**: Saara Nolvi, Kari Rantavuori, Hasse Karlsson, Linnea Karlsson; **Project administration**: Hasse Karlsson, Linnea Karlsson.

## CONFLICTS OF INTEREST

The authors declare no conflicts of interest.

## References

[1] Liinavuori A , Tolvanen M , Pohjola V , Lahti S . Changes in dental fear among Finnish adults: a national survey. Community Dent Oral Epidemiol. 2016;44:128–34. 26482701 10.1111/cdoe.12196

[2] Liinavuori A , Tolvanen M , Pohjola V , Lahti S . Longitudinal interrelationships between dental fear and dental attendance among adult Finns in 2000–2011. Community Dent Oral Epidemiol. 2019;47:309–15. 30941810 10.1111/cdoe.12458

[3] Pohjola V , Lahti S , Vehkalahti MM , Tolvanen M , Hausen H . Association between dental fear and dental attendance among adults in Finland. Acta Odontol Scand. 2007;65:224–30. 17762985 10.1080/00016350701373558

[4] Pohjola V , Lahti S , Vehkalahti MM , Tolvanen M , Hausen H . Age‐specific associations between dental fear and dental condition among adults in Finland. Acta Odontol Scand. 2008;66:278–85. 18720054 10.1080/00016350802293960

[5] Pohjola V , Lahti S , Suominen‐Taipale L , Hausen H . Dental fear and subjective oral impacts among adults in Finland. Eur J Oral Sci. 2009;117:268–85. 19583754 10.1111/j.1600-0722.2009.00631.x

[6] Armfield JM . What goes around comes around: revisiting the hypothesized vicious cycle of dental fear and avoidance. Community Dent Oral Epidemiol. 2013;41:279–87. 23004917 10.1111/cdoe.12005

[7] Pohjola V , Mattila AK , Joukamaa M , Lahti S . Anxiety and depressive disorders and dental fear among adults in Finland. Eur J Oral Sci. 2011;119:55–60. 10.1111/j.1600-0722.2010.00795.x 21244512

[8] Aartman IHA , de Jongh A , van der Meulen MJ . Psychological characteristics of patients applying for treatment in a dental fear clinic. Eur J Oral Sci. 1997;105:384–8. 9395098 10.1111/j.1600-0722.1997.tb02134.x

[9] Locker D , Shapiro D , Liddell A . Overlap between dental anxiety and blood‐injury fears: Psychological characteristics and response to dental treatment. Behav Res Ther. 1997;35:583–90. 9193122 10.1016/s0005-7967(97)00016-8

[10] De Jongh A , Bongaarts G , Vermeule I , Visser K , De Vos P , Makkes P . Blood‐injury‐injection phobia and dental phobia. Behav Res Ther. 1998;36:971–82. 9714947 10.1016/s0005-7967(98)00064-3

[11] Boman UW , Lundgren J , Berggren U , Carlsson SG . Psychosocial and dental factors in the maintenance of severe dental fear. Swed Dent J. 2010;34:121–7. 21121411

[12] Hakeberg M , Hägglin C , Berggren U , Carlsson SG . Structural relationships of dental anxiety, mood, and general anxiety. Acta Odontol Scand. 2001;59:99–103. 11370758 10.1080/000163501750157252

[13] Hägglin C , Hakeberg M , Hällström T , Berggren U , Larsson L , Waern M , et al. Dental anxiety in relation to mental health and personality factors. A longitudinal study of middle‐aged and elderly woman. Eur J Oral Sci. 2001;109:27–33. 11330931 10.1034/j.1600-0722.2001.00946.x

[14] Pekkan G , Kilicoglu A , Hatipoglu H . Relationship between dental anxiety, general anxiety level and depression in patients attending a university hospital dental clinic in Turkey. Community Dent Health. 2011;28:149–53. 21780354

[15] Bernson JM , Elfström ML , Hakeberg M . Dental coping strategies, general anxiety, and depression among adult patients with dental anxiety but with different dental‐attendance patterns. Eur J Oral Sci. 2013;121:270–6. 23659261 10.1111/eos.12039

[16] Halonen H , Salo T , Hakko H , Räsänen P . The association between dental anxiety, general clinical anxiety and depression among Finnish university students. Oral Health Dent Manage. 2014;13:320–5. 24984641

[17] Tellez M , Kinner DG , Heimberg RG , Lim S , Ismail AI . Prevalence and correlates of dental anxiety in patients seeking dental care. Community Dent Oral Epidemiol. 2015;43:135–42. 25346261 10.1111/cdoe.12132

[18] Lahti S , Tolvanen M , Humphris G , Freeman R , Rantavuori K , Karlsson L , et al. Association of depression and anxiety with different aspects of dental anxiety in pregnant mothers and their partners. Community Dent Oral Epidemiol. 2020;48:137–42. 31809556 10.1111/cdoe.12511

[19] Hagqvist O , Tolvanen M , Rantavuori K , Karlsson L , Karlsson H , Lahti S . Changes in dental fear and its relations to anxiety and depression in the FinnBrain Birth Cohort Study. Eur J Oral Sci. 2020;128:429–35. 32875623 10.1111/eos.12736

[20] Lahti SM , Tolvanen MM , Humphris G , Freeman R , Rantavuori K , Karlsson L , et al. Association of depression and anxiety with different aspects of dental anxiety in pregnant mothers and their partners. Community Dent Oral Epidemiol. 2020;48:137‐42. 31809556 10.1111/cdoe.12511

[21] Viinikangas A , Lahti S , Tolvanen M , Freeman R , Humphris G , Joukamaa M . Dental anxiety and alexithymia: gender differences. Acta Odontol Scand. 2009;67:13–8. 18932042 10.1080/00016350802459264

[22] Pohjola V , Mattila A , Joukamaa M , Lahti S . Dental fear and alexithymia among adults in Finland. Acta Odontol Scand. 2011;69:243–7. 21294672 10.3109/00016357.2011.554861

[23] Karukivi M , Suominen A , Scheinin NM , Li R , Ahrnberg H , Rantavuori K , et al. Gender‐specific associations between the dimensions of alexithymia personality trait and dental anxiety in parents of the FinnBrain Birth Cohort Study. Eur J Oral Sci. 2022;130:e12830. 34761432 10.1111/eos.12830

[24] Locker D , Poulton R , Thomson WM . Psychological disorders and dental anxiety in a young adult population. Community Dent Oral Epidemiol. 2001;29:456–63. 11784289 10.1034/j.1600-0528.2001.290607.x

[25] Armfield JM . Cognitive vulnerability: a model of the etiology of fear. Clin Psychol Rev. 2006;26:746–68. 16806621 10.1016/j.cpr.2006.03.007

[26] Locker D , Liddell A , Shapiro D . Diagnostic categories of dental anxiety: a population‐based study. Behav Res Ther. 1999;37:25–37. 9922555 10.1016/s0005-7967(98)00105-3

[27] Rothbart MK , Bates JE . Temperament. In: Damon W , Lerner R , Eisenberg N , eds. Handbook of Child Psychology: *Vol*. 3. Social, emotional, and personality development (6th ed), 2006; pp. 99–166.

[28] Evans D , Rothbart M . Developing a model for adult temperament. J Res Personality. 2007;41:868–88.

[29] Komasi S , Rezaei F , Hemmati A , Rahmani K , Aminato F , Miettinen J . J Int Med Res. 2022;50:1‐27. 10.1177/03000605211070766 PMC8743952 34994240

[30] Kampman O , Viikki M , Leinonen E . Anxiety disorders and temperament‐an update review. Curr Psychiatry Rep. 2017;19:27. 10.1007/s11920-017-0779-5 28417269

[31] Gustafsson A , Broberg A , Bodin L , Berggren U , Arnrup K . Dental behaviour management problems: the role of child personal characteristics. Int J Paediatr Dent. 2010;20:242–53. 20536585 10.1111/j.1365-263X.2010.01046.x

[32] Stenebrand A , Wide Boman U , Hakeberg M . Dental anxiety and temperament in 15‐year olds. Acta Odontol Scand. 2013;71:15–21. 22214361 10.3109/00016357.2011.645068

[33] Bergdahl M , Bergdahl J . Temperament and character personality dimensions in patients with dental anxiety. Eur J Oral Sci. 2003;111:93–8. 12648259 10.1034/j.1600-0722.2003.00028.x

[34] Ludgren J , Elfström M , Berggren U . The relationship between temperament and fearfulness in adult dental phobic patients. Int J Paediatr Dent. 2007;17:460–8. 17935599 10.1111/j.1365-263X.2007.00880.x

[35] Halonen H , Salo T , Hakko H , Räsänen P . Association of dental anxiety to personality traits in a general population sample of Finnish university students, Acta Odontol Scand.2012;70:96–100 21736513 10.3109/00016357.2011.598182

[36] Karlsson L , Tolvanen M , Scheinin NM , Uusitupa HM , Korja R , Ekholm E , et al. FinnBrain Birth Cohort Study Group . Cohort Profile: The FinnBrain Birth Cohort Study (FinnBrain). Int J Epidemiol. 2018;47:1–12. 29025073 10.1093/ije/dyx173

[37] Humphris G , Crawford JR , Hill K , Gilbert A , Freeman R . UK population norms for the modified dental anxiety scale with percentile calculator: adult dental health survey 2009 results. BMC Oral Health. 2013;13:29. 10.1186/1472-6831-13-29 23799962 PMC3691614

[38] Humphris GM , Freeman R , Campell J , Tuutti HD , Souza V . Further evidence for the reliability and validity of the Modified Dental Anxiety Scale. Int Dent J. 2000;50:367–70. 11197195 10.1111/j.1875-595x.2000.tb00570.x

[39] Newton JT , Edwards JC . Psychometric properties of the modified dental anxiety scale: an independent replication. Community Dent Health. 2005;22:40–2. 15819115

[40] Derogatis LR , Lipman RS , Covi L . SCL‐90: an outpatient psychiatric rating scale—preliminary report. Psychopharmacol Bull. 1973;9:13–28. 4682398

[41] Holi MM , Sammallahti PR , Aalberg VA . A Finnish validation study of the SCL‐90. Acta Psychiatr Scand. 1998;97:42–6. 9504702 10.1111/j.1600-0447.1998.tb09961.x

[42] Prinz U , Nutzinger DO , Schulz H , Petermann F , Braukhaus C , Andreas S . Comparative psychometric analyses of the SCL‐90‐R and its short versions in patients with affective disorders. BMC Psychiatry. 2013;13:104. 10.1186/1471-244X-13-104 23537095 PMC3626675

[43] Cox JL , Holden JM , Sagovsky R . Detection of postnatal depression. Development of the 10‐item Edinburgh Postnatal Depression Scale. Br J Psychiatry. 1987;150:782–6. 3651732 10.1192/bjp.150.6.782

[44] Eberhard‐Gran M , Eskild A , Tambs K , Opjordsmoen S , Samuelsen SO . Review of validation studies of the Edinburgh Postnatal Depression Scale. Acta Psychiatr Scand. 2001;104:243–9. 11722298 10.1034/j.1600-0447.2001.00187.x

[45] Matthey S , Barnett B , Kavanagh DJ , Howie P . Validation of the Edinburgh Postnatal Depression Scale for men, and comparison of item endorsement with their partners. J Affect Disord. 2001;64:175–84. 11313084 10.1016/s0165-0327(00)00236-6

[46] Massoudi P , Hwang CP , Wickberg B . How well does the Edinburgh Postnatal Depression Scale identify depression and anxiety in fathers? A validation study in a population based Swedish sample. J Affect Disord. 2013;149:67–74. 23499163 10.1016/j.jad.2013.01.005

[47] Tehseen S , Ramayah T , Salijan S . Testing and controlling for common methods variance: a review of available methods. J Management Sci. 2017;4:142–68.

[48] Erickson K , Dreverts W , Schulkin J . Clucorticoid regulation of diverse cognitive functions in normal and pathological emotional states. Neurosci Biobehav Rev. 2003;27:233‐46. 12788335 10.1016/s0149-7634(03)00033-2

[49] Viitaniemi H , Suominen A , Karlsson L , Mustonen P , Kortesluoma S , Rantavuori K , et al. Hair cortisol concentrations are associated with dental anxiety during pregnancy. Dent J. 2021;9:42. 10.3390/dj9040042 PMC8069593 33920415

[50] Thomson WM , Poulton RG , Kruger E , Davies S , Brown RH , Silva PA . Changes in self‐reported dental anxiety in New Zealand adolescents from ages 15 to 18 years. J Dent Res. 1997;76:1287‐91. 9168862 10.1177/00220345970760060801

[51] Helkimo AN , Rolander B , Koch G . Dental fear in school children and young adults attending public dental health care: prevalence and relationship to gender, oral disease and dental treatment; trends over 40 years. BMC Oral Health. 2022;22:146. 10.1186/s12903-022-02166-6 35473601 PMC9044703

[52] Armfield JM , Heaton LJ . Management of fear and anxiety in the dental clinic: a review. Aust Dent J. 2013;58:390‐407. 24320894 10.1111/adj.12118

